# Advances in Irradiated Livestock Vaccine Research and Production Addressing the Unmet Needs for Farmers and Veterinary Services in FAO/IAEA Member States

**DOI:** 10.3389/fimmu.2022.853874

**Published:** 2022-03-28

**Authors:** Hermann Unger, Richard T. Kangethe, Fatima Liaqat, Gerrit J. Viljoen

**Affiliations:** Animal Production and Health Section, Joint FAO/IAEA Centre of Nuclear Techniques in Food and Agriculture, Department of Nuclear Sciences and Applications, International Atomic Energy Agency (IAEA), Vienna, Austria

**Keywords:** irradiated vaccines, FAO/IAEA, coordinated research projects (CRP), member states (MS), livestock

## Abstract

The Animal Production and Health section (APH) of the Joint FAO/IAEA Centre of Nuclear Techniques in Food and Agriculture at the International Atomic Energy Agency has over the last 58 years provided technical and scientific support to more than 100 countries through co-ordinated research activities and technical co-operation projects in peaceful uses of nuclear technologies. A key component of this support has been the development of irradiated vaccines targeting diseases that are endemic to participating countries. APH laboratories has over the last decade developed new techniques and has put in place a framework that allows researchers from participating member states to develop relevant vaccines targeting local diseases while using irradiation as a tool for improving livestock resources.

## Introduction

**Figure 1:**
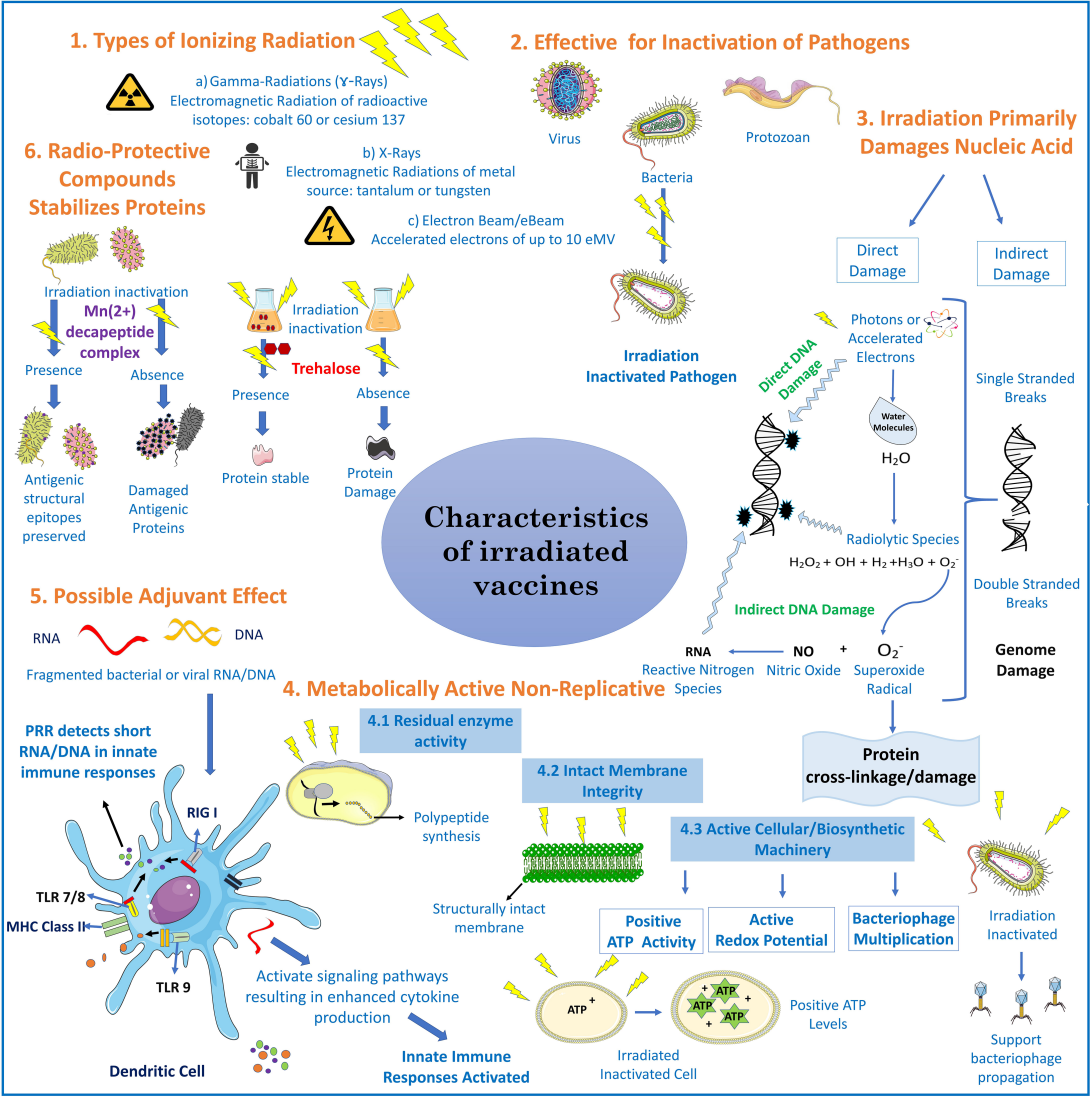
Characteristics of irradiated vaccines: Irradiated vaccines are produced mainly by delivering ionizing radiation through gamma ray, X-ray, or electronic beam (e-Beam) irradiation (13). The mechanism behind inactivation of pathogens is through direct and indirect damage of genetic material (DNA/RNA) and cross linking of proteins. Direct genome damage is by contact of photons (from gamma-rays/X-rays) or accelerated electrons (10eMV mega electron volt) through the breakage of phosphodiester bonds (14). Indirect damage is by highly reactive short lived radiolytic species such as hydrogen peroxide (H_2_O_2_), hydroxyl radical, hydrogen (H_2_) and superoxide radicals $\left({\text{O}}_{2}^{-}\right)$ , that are the product of endogenous water molecules radiolysis. ${\text{O}}_{2}^{-}$ can cause protein cross linkage and upon its reaction with nitric oxide it can generate reactive nitrogen species (RNS) that damages nucleic acids (15). In bacteria and parasites, irradiation stops replication yielding metabolically active organisms that present structural antigens and functional proteins in a vaccine as reported through ATP production, redox potential, or bacteriophage multiplication (16). Irradiation produces short RNA/DNA fragments which activate pathogen pattern recognition receptors (PRR)s, for example retinoic acid-inducible gene I (RIG I) or toll like receptors (TLR’s) in the innate immune system thus acting as a vaccine adjuvant (12). Adding radio-protective compounds such Mn2+-decapeptide complex (MDP) derived from *Deinococcus radiodurans* or trehalose preserves immunogenic epitopes (10, 11).

Vaccines are a mainstay in supporting livestock health both in intensive industrial based animal systems and in the pastoralist livestock industry where they play a crucial role in supporting vulnerable communities. The development of livestock vaccines fits well within the framework of the Sustainable Development Goals specifically SD Goal 2 that aims to end hunger, achieve food security, improve nutrition, and promote sustainable agriculture (1). There are 117 OIE-listed diseases and many of these could be better addressed by a vaccine for control or require an improvement in the current vaccine setup (2, 3). In 2011, FAO declared the eradication of rinderpest globally which was achieved with the use of an attenuated live vaccine, thus emphasizing the importance of livestock vaccines in agriculture (4). The animal health and production laboratory (APHL), a section of the Joint FAO/IAEA center based at Seibersdorf, was involved in sero-monitoring of the Rinderpest vaccination programme and supported the development and validation of diagnostic tests that correlated antibody status with animal or herd level protection (5). This participation led to increasing the activities of the laboratory in different aspects of veterinary vaccine production with the use of irradiation as a tool for researching new vaccine formulations and in serological surveillance for disease eradication programs. Irradiation has previously been used as a technique to address some of the gaps that exist in developing livestock vaccines but was later abandoned for newer techniques such as recombinant and gene-based vaccines (6). There has only ever been one irradiated livestock vaccine in common use for the cattle lung nematode *Dictyocaulus viviparous* that utilises irradiated L3 stage larvae for vaccination (7–9). Other diseases were not pursued further due to the lack of adequate immunological tools that could assess the effect of using irradiated vaccines. With more recent advances in livestock immunology, there has been a chance to re-examine irradiation for vaccine development with a novel approach targeting replication deficiency while maintaining some metabolic activities and reducing conformational alterations of antigens by employing new radio-protectant compounds such as manganese ions (Mn2^+^) and Trehalose (10, 11). Additional functions for irradiated material have also been explored e.g., as adjuvants (12). A comprehensive summary of the characteristics of irradiated vaccines is found on **Figure 1**.

## Background on Irradiated Vaccines

Although live attenuated vaccines have been successfully used in preventing diseases, they can trigger side effects in recipients, and in the case of viruses, revert back to infectivity (17, 18). Chemically inactivated and recombinant vaccines are however considered safe but unfortunately are often unable to elicit an effective immune response that is protective in all vaccinated individuals e.g. chemically inactivated vaccines against seasonal flu have an efficacy of only 30- 40% among the elderly (19). Irradiation therefore offers an alternative that can be as effective as live attenuated vaccines yet equally safe as killed or recombinant vaccines (20). The use of irradiation for vaccine development was initiated almost a century ago as an alternative to live attenuated and chemically inactivated *Shigella spp* bacteria (21). In livestock, irradiation experiments using the isotope Cobalt-60 (Co-60) were carried out in the late sixties to study *Trypanosome* spp., the causative agent of Nagana in livestock (22–27). Many of these experiments used higher than necessary irradiation doses to kill their targets rather than rendering them non-infective. Subsequent developments in immunology that described killed but metabolically active bacteria led to the idea that metabolic products produced by living but non-replicating irradiated pathogens made superior antigens compared to those produced by traditional chemical inactivation techniques (17, 18, 28, 29). Irradiation, when compared to chemical methods, is a rapid method of inactivation that requires no post inactivation manipulation and is suitable for industrial production (30). Exposure to radiation randomly causes breaks in single and double stranded nucleic acids that most cellular systems cannot repair, thus eliminating the possibility of reversion back to a virulent state (31). Radiation mediated genetic damage is also comparatively more severe when compared to chemical inactivation (32, 33). The ROS (reactive oxygen species) generated during the inactivation process, whether chemical or irradiation, imparts additional indirect nucleic acid and protein damage (31). Chemical inactivation however, possess the challenge of ineffective membrane penetration by chemical agents and residues in the products that must be eliminated by expensive and time consuming down-stream purification steps (34). The bigger the protein damage during inactivation, the less specific and immunogenic the vaccine becomes due to the loss of conformational epitopes. Epitope damage is more severe for chemically inactivated pathogens when compared to irradiated ones due to radio-protectants employed thus eliciting better responses as has been observed with the Gamma flu vaccine (34, 35). The quality of antigens used for immunization becomes especially important when targeting intracellular parasites where humoral responses have limited efficacy. During *L. monocytogenes* infections, specific neutralizing antibodies fail to clear intracellular infection which is vital to establish infection in the host (36). The recruitment of MHC class I mediated CD8 T cells is necessary for pathogen elimination and can only be induced by vaccines that mimic a natural infection (29). Vaccination with irradiated and killed but metabolically active *Listeria* spp. is able to elicit this crucial response when compared to chemically treated Listeria (17, 37). Using a wider repertoire of conformational epitopes that retain their secondary structures after irradiation becomes even more crucial in diseases where the correlates of immunity are unknown or poorly understood as neutralizing antibodies are not always a marker for protective immunity. The required type of immune response elicited by any radio-vaccine ultimately depends on the pathology pathway in the host vaccinated as it should ultimately strive to mimic the wild-type situation without replication. In the case of bacterial infections, irradiated *Salmonella* elicit T-independent immune protection through both humoral responses (IgG2b, IgG3) and CD4^+^ T-cell mediated responses (Th1, Th17) (38). Numerous other bacterial and parasitic pathogens have been irradiated for vaccine development and are in various stages of vaccine development. These include pathogens such as *E. coli, Brucella, Clostridium, Mycobacterium, Plasmodium, Toxoplasma*, *Ancylostoma*, and *Schistosoma* all observed to be non-dividing but metabolically active after irradiation treatment (16, 39–45). It is clear from these experiments that irradiation generates metabolically active but non-replicative pathogens mainly for bacteria and protozoans, where they resemble a live infective pathogen more closely.

The approach for inactivating viruses using irradiation is however considered different. Viruses, obligate intracellular pathogens, are metabolically dependent on their host for viral replication and reproduction (46). An inactivated virus would essentially be unable to replicate within the host cell even after gaining entry. Gamma inactivated influenza A (γ-flu) can elicit IFN-I dependent partial lymphocyte activation *in vivo* contrary to UV and formalin treated vaccines. This is associated with the synthesis of structural internal viral proteins such as nucleoproteins in the cytosol of antigen presenting cells (47). The IFN-I response elicited by γ-flu can be attributed to the preservation of conformational peptides that are presented *via* MHC class I which trigger a type 1 response that is absent in formulations prepared using formalin or UV. Preparations made with formalin lose peptide moieties that elicit a cell mediated inflammatory response but still maintain humoral responses which are not effective at preventing disease (19). Given that irradiation leads to major nucleic acid damage when compared to other techniques, the risk of reversion in pathogens with segmented genomes is low. Innocuity testing post irradiation is however required for all formulations before further use to confirm inactivation. In the case of Avian influenza, this is carried out using embryonated chicken eggs which are susceptible to infection and are routinely used for virus isolation during surveillance of the disease (48). Other livestock viruses that have been subjected to irradiation studies with great promise include Bluetongue, Equine encephalitis and rabies amongst others (49–51).

Irradiation has also been used to improve inactivated vaccines from toxin producing pathogens. The best way to currently prevent tetanus is through vaccination using formaldehyde inactivated tetanus toxin (52). In order to overcome the disadvantages of exposing vaccine recipients to long term effects of formaldehyde and associated salts, irradiation was used to inactivate tetanus toxin (53). The toxin was inactivated at 5 kGy but retained immunogenicity at 8 KGy which was the upper limit of irradiation used in the experiment. In addition, pure irradiated toxin retained more than 50% of its enzymatic activity. Future studies will optimize the production process, detoxification and explore its feasibility as an adjuvant (53). Other toxin producing pathogens tested in irradiation studies include *Mycobacterium* spp., Anthrax, Cholera, Coli and paratyphoid B where irradiation does not necessarily inactivate the toxins in contaminated meat (28, 54–56). Irradiation has also been used in the research and production of several snake venom vaccines including African elapid, viperid and Crotalus venoms (57, 58). There are currently no effective treatments or vaccines against prion diseases due to their complex biology (59, 60). Radiation induced protein damage is considered a sterilization method of infectious proteins like prions in aqueous solution and the inactivation of infectious scrapie from transmissible spongiform encephalopathy (33, 61, 62). It was noted that high doses of up to 100 KGy were not enough to completely inactivate prions but instead reduced their quantities by 4 – 5 logs. Diluting the original stock of prion prepared had a stronger effect on reducing the chances of causing disease in mice when compared to irradiation (63). A combination of dilution and irradiation would be considered the best approach to developing antigens for anti-prion vaccines.

## Technical Support to Member States Through Coordinated Research Activities

Due to the requirement for basic level research in developing new irradiation vaccine formulations, APHL has initiated several different coordinated research programs (CRP) and technical cooperation projects (TCP) that have run concurrently since 2009 (**Supplementary Table 1**). The initial research project required participating members to establish the basic parameters required to carry out experiments with their chosen diseases. The participants were requested to devise a work plan that included the following points of interest for each disease in the CRP for future experiments.

1. To establish a dose of irradiation for attenuation that is consistent in scale i.e., using KGy as opposed to Krad, due to inconsistencies in groups studying the same pathogen.
2. To determine indicator/s of attenuation of the pathogen to be used for vaccination
3. To describe the representative animals used in vaccine experiments and determine the appropriate sample size.
4. To describe the parameters for vaccination including the amount of pathogen used and number of times and period of duration between inoculations.
5. To describe the parameters for challenge including number of non-irradiated pathogens used, duration of challenge after vaccination and the difference between homologous Vs heterologous.
6. To establish the criteria for protection i.e., full protection or alleviation of pathology associated with the pathogen and parameters to be measured after challenge.
7. To establish a sequence of events starting with safety at dose of irradiation, diagnostic tools available for measuring protection and the performance of the vaccine generated.
8. To identify the immune response important for protection where possible.

As a result of these activities, various basic parameters were established at the end of the first two CRP as shown in **Table 1**. To support participating laboratories further with ongoing activities, APH laboratories also developed tools that could be used in evaluating vaccine efficacy. Quantitative PCR panels that measure innate and adaptive immunity were developed for ruminants, pigs and chicken (70). Quantitative PCR panels are easy to adopt especially where collaborating partners have limited resources to carry out other assays. Similar panels using flow cytometry, ELISA, ELISPOT, Immunofluorescence, microarray and RNAseq technologies are also currently under development. A more complex assay that measures vaccine immunogenicity *in vitro* using bovine monocyte derived dendritic cells was also developed for use as a filter for antigens before proceeding to animal experiments (71). This would be particularly useful in cases where the number of irradiated vaccine candidates was large with limited animal testing facilities.

**Table 1:** Comparison of different irradiation experiments carried out by IAEA and partners.

Species	Strain	Disease	Administered Deactivation dose (KGy)	D10 (KGy)	Post irradiation activity	*In vivo* innoculation/Challenge	Notes
***Brucella abortus***	S19	Abortion in pregnant cattle	3.5	NA	alamarBlue^®^,	1x10^7^/S2308 strain	Murine Macrophage infection assays
***Brucella melitensis***	Rev1	Human and bovine disease (zoonotic)	1 - 5	NA	alamarBlue^®^	NA	cross-species irradiated vaccine?
***Theileria annulate***	local strain/Schizont stage vaccine	Theileriosis in ruminants	0.15 - 0.4	NA	NA	NA	To replace schizont stage vaccine, 0.4 KGy used for irradiating blood with 21% parasitaemia (10ml/calf)
***Fasciola hepatica***	Local strain	Common liver fluke (zoonotic)	3 - 24	NA	NA	NA	(64)
***Fasciola gigantica***	local strain	Tropical liver disease (zoonotic)	0.030-0.050	NA	NA	metacercaria; 40/oral dose	
***Haemonchus contortus***	local strain	Blood feeding nematodes for sheep and goat	0.17 - 170	NA	NA	10.000 larvae	Larvae stage III; 99% protection;
***Ichthyophthirius multifiliis***	local strain	Protozoan ecto-parasite in fish	1.5	5.2	Lysozyme, alkaline phosphate, protease and Estarases activitiy	100 trophonts/10 fish	(65)
***Trypanosoma evansi***	RoTat 1.2	Mechanically transmitted blood protozoan parasite	0.2	0.1983	CFSE (replication), Parasite growth	1x 10^4^/10^3^ homologous & heterologous Can86K	virulence gene mining
***African Swine fever***	Estonia 124	African swine fever	30	1.81	NA	10^7.25^HAU/heterologous	No protection
						Armenia 2008	
***Avian Influenza virus***	H9N2	Avian influenza	60	5.46	Hemagglutination assay, inoculation in embryonated eggs	128 HAU/10^3^,10^4^ & 10^6^	Protection at lower doses with oral-nasal application
***Avian pathogenic Escherichia coli (APEC)***	APEC	colibacillosis	1.2	0.89	NA	NA	Ongoing
***Lumpy skin disease virus (LSDV)***	Various	Lumpy skin disease	30	3.75	NA	NA	Ongoing
***Theileria parva***	local strain	East Coast fever	0.9	NA	NA	NA	ongoing
***Avian Influenza virus***	H9N2	LPAI	29.52 (frozen)	3.36	NA	NA	(66)
***M. haemolytica***	local from pneumonic lungs	Pneumonic mannheimiosis	2-20	NA	NA	2×10^10^/3.6 x10^10^	(67)
***Salmonella gallinarum***	Field strain	Fowl typhoid	2.4 (RT)	NA	NA	10^8^	(68)
***White spot syndrome virus***	Local	White spot syndrom	15	2.56	NA	NA	(69)
***Foot-and-mouth disease virus***	Local strain IRN/1/2007	Foot-and-mouth disease	50	4.8	NA	NA	(69)
***P. multocida***	Local (MK802880, NVI)	Fowl cholera	1	NA	NA	NA	(55)

*NA (Not Available).

## Future Perspectives

The future for developing irradiated vaccines in veterinary medicine is bright. Recent advances in delivering ionising radiation using safer methods other than Co-60 have greatly advanced with the development of inactivation techniques like low energy electron beam irradiation that maintains antigenicity for Influenza A (H3N8), Porcine reproductive and respiratory syndrome (PRRSV), Equine herpes (EHV-1), Zika, Respiratory syncytial virus, *Rodentibacter pneumotropicus*, *Bacillus cereus* and *E. coli* (72–74). Irradiated pathogens have also been used as adjuvants as in the case of gamma irradiated influenza A virus co-administered with Semliki Forest virus where it displayed the potential to enhance immune response against Semliki Forest virus by six-fold in mouse (12). This adjuvant activity is attributed to γ-irradiated influenza A virus which behaves like Poly I:C (synthetic dsRNA) and elicits an interferon type I (IFN-I) humoral response through TLR3 (toll like receptor 3) signaling plus IFN-I mediated lymphocytes activation (12, 75). Irradiated parasite vaccines have also opened new areas of immunological study, as in the case of irradiated *Salmonella gallinarum* protecting mice and chicken from infection and *Haemonchus contortus* where metabolically active irradiated larvae of parasites remain immobilised in the abomasum of vaccinated sheep conferring long term protective response and long term immune stimulation (38, 64). The introduction or generation of unmethylated cytosine–guanine dinucleotide (CpGs) during irradiation and the application of such vaccines address mucosal immunity and inoculation strategies which are desirable when dealing with intensive farming systems (76). Extensive epitope damage due to high irradiation doses has been mitigated with the development of radio protective compounds such as manganese ions (Mn2^+^) and Trehalose which reduce structural damage of surface epitopes (10, 11).

In summary, recent research over the past 10 years has created a new base for the rational development of irradiated vaccines. New irradiation devices like x-rays or e-beams which do not need special radiation protection and are economically viable can be installed in bio-safety laboratories (73). A broad spectrum of molecular tests replaces traditional cell based immune assays that require expensive equipment and expertise, and the *in vitro* evaluation of immune induction replaces animal experiments where possible (70, 71). This research can effectively be carried out on local diseases in countries that have previously relied on results from advanced laboratories that increasingly cannot prioritise them due to constrains on funding and human resource capacities.

## Author Contributions

All authors listed have made a substantial, direct, and intellectual contribution to the work, and approved it for publication.

## Conflict of Interest

The authors declare that the research was conducted in the absence of any commercial or financial relationships that could be construed as a potential conflict of interest.

## Publisher’s Note

All claims expressed in this article are solely those of the authors and do not necessarily represent those of their affiliated organizations, or those of the publisher, the editors and the reviewers. Any product that may be evaluated in this article, or claim that may be made by its manufacturer, is not guaranteed or endorsed by the publisher.

## References

[1] United Nations. Education | Department of Economic and Social Affairs. Sustain Dev (2021) 28–9.

[2] Meeusen ENT Walker J Peters A Pastoret PP Jungersen G. Current Status of Veterinary Vaccines. Clin Microbiol Rev (2007) 20:489–510. doi: 10.1128/CMR.00005-07 17630337 PMC1932753

[3] Available at: http://www.animalhealthsurveillance.agriculture.gov.ie/oielisteddiseases/ (date accessed 11/01/2022).

[4] OIE. Resolution No. 18: Declaration of Global Eradication of Rinderpest and Implementation of Follow-Up Measures to Maintain World Freedom From Rinderpest. Paris: 79th Session of the World Assembly of Delegates. (2011).

[5] Njeumi F Taylor W Diallo A Miyagishima K Pastoret PP Vallat B. The Long Journey: A Brief Review of the Eradication of Rinderpest. OIE Rev Sci Tech (2012) 31:729–46. doi: 10.20506/rst.31.3.2157 23520729

[6] Poulet H Minke J Pardo MC Juillard V Nordgren B Audonnet JC. Development and Registration of Recombinant Veterinary Vaccines. The Example of the Canarypox Vector Platform. Vaccine (2007) 25:5606–12. doi: 10.1016/j.vaccine.2006.11.066 17227690

[7] Strube C Haake C Sager H Schorderet Weber S Kaminsky R Buschbaum S. Vaccination With Recombinant Paramyosin Against the Bovine Lungworm Dictyocaulus Viviparus Considerably Reduces Worm Burden and Larvae Shedding. Parasites Vectors (2015) 8:119. doi: 10.1186/s13071-015-0733-5 25890350 PMC4352246

[8] Jarrett WF Jennings FW Mcintyre WI Mulligan W Thomas BA Urquhart GM. Immunological Studies on Dictyocaulus Viviparus Infection: The Immunity Resulting From Experimental Infection. Immunology (1959) 2:252–61. PMC1423946 14406845

[9] McLeonard C Van Dijk J. Controlling Lungworm Disease (Husk) in Dairy Cattle. In Pract (2017) 39:408–19. doi: 10.1136/inp.j4038

[10] Gaidamakova EK Myles IA McDaniel DP Fowler CJ Valdez PA Naik S. Preserving Immunogenicity of Lethally Irradiated Viral and Bacterial Vaccine Epitopes Using a Radio- Protective Mn2+-Peptide Complex From Deinococcus. Cell Host Microbe (2012) 12:117–24. doi: 10.1016/j.chom.2012.05.011 PMC4073300 22817993

[11] Nair CKK Parida DK Nomura T. Radioprotectors in Radiotherapy. J Radiat Res (2001) 42:21–37. doi: 10.1269/jrr.42.21 11393887

[12] Babb R Chan J Khairat JE Furuya Y Alsharifi M. Gamma-Irradiated Influenza a Virus Provides Adjuvant Activity to a Co-Administered Poorly Immunogenic SFV Vaccine in Mice. Front Immunol (2014) 5:267. doi: 10.3389/fimmu.2014.00267 24959166 PMC4050334

[13] Pillai SD Shayanfar S Venturi M Pillai s-pillai SD. Electron Beam Technology and Other Irradiation Technology Applications in the Food Industry. Top Curr Chem (2016) 375:6. doi: 10.1007/s41061-016-0093-4 28000138

[14] Miller RB. Electronic Irradiation of Foods. Boston, MA: Springer US (2005). doi: 10.1007/0-387-28386-2

[15] Tahergorabi R Matak KE Jaczynski J. Application of Electron Beam to Inactivate Salmonella in Food: Recent Developments. Food Res Int (2012) 45:685–94. doi: 10.1016/j.foodres.2011.02.003

[16] Hieke ASC Pillai SD. Escherichia Coli Cells Exposed to Lethal Doses of Electron Beam Irradiation Retain Their Ability to Propagate Bacteriophages and are Metabolically Active. Front Microbiol (2018) 9:2138. doi: 10.3389/fmicb.2018.02138 30250460 PMC6139317

[17] Brockstedt DG Bahjat KS Giedlin MA Liu W Leong M Luckett W. Killed But Metabolically Active Microbes: A New Vaccine Paradigm for Eliciting Effector T-Cell Responses and Protective Immunity. Nat Med (2005) 11:853–60. doi: 10.1038/nm1276 16041382

[18] Dubensky TW Jr. Skoble J Lauer P Brockstedt DG Dubensky TW Skoble J. Killed But Metabolically Active Vaccines. Curr Opin Biotechnol (2012) 23:917–23. doi: 10.1016/J.COPBIO.2012.04.005 22608846

[19] Furuya Y. Return of Inactivated Whole-Virus Vaccine for Superior Efficacy. Immunol Cell Biol (2012) 90:571–8. doi: 10.1038/icb.2011.70 21844883

[20] Seo HS. Application of Radiation Technology in Vaccines Development. Clin Exp Vaccine Res (2015) 4:145. doi: 10.7774/cevr.2015.4.2.145 26273573 PMC4524899

[21] Moore HN Kersten H. Preliminary Note on the Preparation of Non-Toxic Shiga Dysentery Vaccines by Irradiation With Soft X-Rays. J Bacteriol (1936) 31:581–4. doi: 10.1128/jb.31.6.581-584.1936 PMC543749 16559915

[22] Duxbury RE Sadun EH. Resistance Produced in Mice and Rats by Inoculation With Irradiated Trypanosoma Rhodesiense. J Parasitol (1969) 55:859–65. doi: 10.2307/3277231 4980777

[23] Duxbury RE Anderson JS Wellde BT Sadun EH Muriithi IE. Trypanosoma Congolense: Immunization of Mice, Dogs, and Cattle With Gamma-Irradiated Parasites. Exp Parasitol (1972) 32:527–33. doi: 10.1016/0014-4894(72)90071-9 4648294

[24] Duxbury RE Sadun EH Anderson JS. Immunization of Monkeys Against a Recently Isolated Human Strain of Trypanosoma Rhodesiense by Use of Gamma Irradiation. Trans R Soc Trop Med Hyg (1973) 67:266–7. doi: 10.1016/0035-9203(73)90172-7 4206107

[25] Wellde BT Duxbury RE Sadun EH Langbehn HR Lötzsch R Deindl G. Experimental Infections With African Trypanosomes: IV. Immunization of Cattle With Gamma-Irradiated Trypanosoma Rhodesiense. Exp Parasitol (1973) 34:62–8. doi: 10.1016/0014-4894(73)90063-5 4722486

[26] Sadun EH Johnson AJ Nagle RB Duxbury RE. Experimental Infections With African Trypanosomes. V. Preliminary Parasitological, Clinical, Hematological, Serological, and Pathological Observations in Rhesus Monkeys Infected With Trypanosoma Rhodesiense. Am J Trop Med Hyg (1973) 22:323–30. doi: 10.4269/ajtmh.1973.22.323 4196287

[27] Duxbury RE Sadun EH West JE. Relative Effectiveness of Neutron and Gamma Radiation of Trypanosomes for Immunizing Mice Against African Trypanosomiasis. Trans R Soc Trop Med Hyg (1975) 69:484–5. doi: 10.1016/0035-9203(75)90104-2 1228986

[28] Skoble J Beaber JW Yi G Lovchik JA Sower LE Liu W. Killed But Metabolically Active Bacillus Anthracis Vaccines Induce Broad and Protective Immunity Against Anthrax. Infect Immun (2009) 77:1649–63. doi: 10.1128/IAI.00530-08 PMC2663168 19168734

[29] Frankel FR. Vaccine Wakes From the Dead. Nat Med (2005) 11:833–4. doi: 10.1038/nm0805-833 16079877

[30] Singh A Singh H. Time-Scale and Nature of Radiation-Biological Damage: Approaches to Radiation Protection and Post-Irradiation Therapy. Prog Biophys Mol Biol (1982) 39:69–107. doi: 10.1016/0079-6107(83)90014-7 7048420

[31] Reisz JA Bansal N Qian J Zhao W Furdui CM. Effects of Ionizing Radiation on Biological Molecules - Mechanisms of Damage and Emerging Methods of Detection. Antioxidants Redox Signal (2014) 21:260–92. doi: 10.1089/ars.2013.5489 PMC4060780 24382094

[32] Olive PL. The Role of DNA Single- and Double-Strand Breaks in Cell Killing by Ionizing Radiation. Radiat Res (1998) 150:S42–51. doi: 10.2307/3579807 9806608

[33] Miekka SI Forng RY Rohwer RG MacAuley C Stafford RE Flack SL. Inactivation of Viral and Prion Pathogens by γ-Irradiation Under Conditions That Maintain the Integrity of Human Albumin. Vox Sang (2003) 84:36–44. doi: 10.1046/j.1423-0410.2003.00256.x 12542732

[34] Alsharifi M Müllbacher A. The γ-Irradiated Influenza Vaccine and the Prospect of Producing Safe Vaccines in General. Immunol Cell Biol (2010) 88:103–4. doi: 10.1038/icb.2009.81 19859081

[35] Chen F Seong Seo H Ji HJ Yang E Choi JA Yang JS. Characterization of Humoral and Cellular Immune Features of Gamma-Irradiated Influenza Vaccine. Hum Vaccin Immunother (2021) 17:485–96. doi: 10.1080/21645515.2020.1780091 PMC7899634 32643515

[36] Jones GS Bussell KM Myers-Morales T Fieldhouse AM Bou Ghanem EN D’Orazio SEF. Intracellular Listeria Monocytogenes Comprises a Minimal But Vital Fraction of the Intestinal Burden Following Foodborne Infection. Infect Immun (2015) 83:3146–56. doi: 10.1128/IAI.00503-15 PMC4496611 26015479

[37] Datta SK Okamoto S Hayashi T Shin SS Mihajlov I Fermin A. Vaccination With Irradiated Listeria Induces Protective T Cell Immunity. Immunity (2006) 25:143–52. doi: 10.1016/j.immuni.2006.05.013 16860763

[38] Ji HJ Byun E-BB Chen F Ahn KB Jung HK Han SH. Radiation-Inactivated S. Gallinarum Vaccine Provides a High Protective Immune Response by Activating Both Humoral and Cellular Immunity. Front Immunol (2021) 12:717556/BIBTEX. doi: 10.3389/FIMMU.2021.717556/BIBTEX 34484221 PMC8415480

[39] Magnani DM Harms JS Durward MA Splitter GA. Nondividing But Metabolically Active Gamma-Irradiated Brucella Melitensis is Protective Against Virulent B. Melitensis Challenge in Mice. Infect Immun (2009) 77:5181–9. doi: 10.1128/IAI.00231-09 PMC2772552 19703982

[40] Bhatia SS. SS. Investigations into Metabolically Active yet Non-Culturable (MAyNC) Clostridium perfringens to Control Necrotic Enteritis in Broiler Chickens. Doctoral dissertation: Texas A&M University (2021). Available at: https://oaktrust.library.tamu.edu/handle/1969.1/193108

[41] Yang JD Mott D Sutiwisesak R Lu YJ Raso F Stowell B. Mycobacterium Tuberculosis-Specific CD4+and Cd8+T Cells Differ in Their Capacity to Recognize Infected Macrophages. PloS Pathog (2018) 14:e1007060. doi: 10.1371/journal.ppat.1007060 29782535 PMC6013218

[42] Luke TC Hoffman SL. Rationale and Plans for Developing a non-Replicating, Metabolically Active, Radiation-Attenuated Plasmodium Falciparum Sporozoite Vaccine. J Exp Biol (2003) 206:3803–8. doi: 10.1242/jeb.00644 14506215

[43] da Costa A Zorgi NE do Nascimento N Galisteo AJ de Andrade HF. Gamma Irradiation of Toxoplasma Gondii Protein Extract Improve Immune Response and Protection in Mice Models. BioMed Pharmacother (2018) 106:599–604. doi: 10.1016/j.biopha.2018.06.155 29990848

[44] Fujiwara RT Loukas A Mendez S Williamson AL Bueno LL Wang Y. Vaccination With Irradiated Ancylostoma Caninum Third Stage Larvae Induces a Th2 Protective Response in Dogs. Vaccine (2006) 24:501–9. doi: 10.1016/j.vaccine.2005.07.091 16140437

[45] El Ridi R Tallima H. Why the Radiation-Attenuated Cercarial Immunization Studies Failed to Guide the Road for an Effective Schistosomiasis Vaccine: A Review. J Adv Res (2015) 6:255–67. doi: 10.1016/j.jare.2014.10.002 PMC4522536 26257924

[46] Chazal N Gerlier D. Virus Entry, Assembly, Budding, and Membrane Rafts. Microbiol Mol Biol Rev (2003) 67:226–37. doi: 10.1128/mmbr.67.2.226-237.2003 PMC156468 12794191

[47] Furuya Y Chan J Wan EC Koskinen A Diener KR Hayball JD. Gamma-Irradiated Influenza Virus Uniquely Induces IFN-I Mediated Lymphocyte Activation Independent of the TLR7/MyD88 Pathway. PloS One (2011) 6:1–12. doi: 10.1371/journal.pone.0025765 PMC3187801 21998693

[48] Woolcock PR McFarland MD Lai S Chin RP. Enhanced Recovery of Avian Influenza Virus Isolates by a Combination of Chicken Embryo Inoculation Methods. Avian Dis (2001) 45:1030–5. doi: 10.2307/1592884 11785874

[49] Campbell CH Barber TL Knudsen RC Swaney LM. Immune Response of Mice and Sheep to Bluetongue Virus Inactivated by Gamma Irradiation. Prog Clin Biol Res (1985) 178:639–47. 2989913

[50] Ceccaldi PE Marquette C Weber P Gourmelon P Tsiang H. Ionizing Radiation Modulates the Spread of an Apathogenic Rabies Virus in Mouse Brain. Int J Radiat Biol (1996) 70:69–75. doi: 10.1080/095530096145346 8691037

[51] Honnold SP Bakken RR Fisher D Lind CM Cohen JW Eccleston LT. Second Generation Inactivated Eastern Equine Encephalitis Virus Vaccine Candidates Protect Mice Against a Lethal Aerosol Challenge. PloS One (2014) 9:e104708. doi: 10.1371/journal.pone.0104708 25116127 PMC4130539

[52] Borella-Venturini M Frasson C Paluan F De Nuzzo D Di Masi G Giraldo M. Tetanus Vaccination, Antibody Persistence and Decennial Booster: A Serosurvey of University Students and at-Risk Workers. Epidemiol Infect (2017) 145:1757–62. doi: 10.1017/S0950268817000516 PMC9203330 28294099

[53] Sartori GP da Costa A dos Santos Macarini FL Mariano DOC Pimenta DC Spencer PJ. Characterization and Evaluation of the Enzymatic Activity of Tetanus Toxin Submitted to Cobalt-60 Gamma Radiation. J Venom Anim Toxins Incl Trop Dis (2021) 27:1–13. doi: 10.1590/1678-9199-JVATITD-2020-0140 PMC8092855 33995513

[54] Cha SB Kim WS Kim JS Kim H Kwon KW Han SJ. Repeated Aerosolized-Boosting With Gamma-Irradiated Mycobacterium Bovis BCG Confers Improved Pulmonary Protection Against the Hypervirulent Mycobacterium Tuberculosis Strain HN878 in Mice. PloS One (2015) 10:e0141577. doi: 10.1371/journal.pone.0141577 26509812 PMC4624807

[55] Dessalegn B Bitew M Asfaw D Khojaly E Ibrahim SM Abayneh T. Gamma-Irradiated Fowl Cholera Mucosal Vaccine: Potential Vaccine Candidate for Safe and Effective Immunization of Chicken Against Fowl Cholera. Front Immunol (2021) 12:768820. doi: 10.3389/fimmu.2021.768820 34917086 PMC8670175

[56] Lawrence EA Duran-Reynals F. The Effect of Combining Bacterial Toxins and X-Ray Irradiation in the Treatment of a Transplantable Mouse Carcinoma. Yale J Biol Med (1941) 14:177–81. PMC2601057 21434005

[57] de la Rosa G Olvera F Cruz E Paniagua D Corzo G. Use of Irradiated Elapid and Viperid Venoms for Antivenom Production in Small and Large Animals. Toxicon (2018) 155:32–7. doi: 10.1016/j.toxicon.2018.10.001 30315836

[58] Clissa PB do Nascimento N Rogero JR. Toxicity and Immunogenicity of Crotalus Durissus Terrificus Venom Treated With Different Doses of Gamma Rays. Toxicon (1999) 37:1131–41. doi: 10.1016/S0041-0101(98)00249-9 10400297

[59] Mabbott NA. Prospects for Safe and Effective Vaccines Against Prion Diseases. Expert Rev Vaccines (2014) 14:1–4. doi: 10.1586/14760584.2015.965691 25266267

[60] Wisniewski T Goñi F. Vaccination Strategies. In: Handbook of Clinical Neurology. Elsevier. p. 419–30. doi: 10.1016/B978-0-444-63945-5.00023-4 29887149

[61] Rohwer RG. Scrapie Infectious Agent is Virus-Like in Size and Susceptibility to Inactivation. Nature (1984) 308:658–62. doi: 10.1038/308658a0 6424032

[62] Latarjet R. Inactivation of the Agents of Scrapie, Ceutzfeldt-Jakob Disease, and Kuru by Radiations. In: Slow Transmissible Diseases of the Nervous System Amsterdam (1979) 2(60):387–408.

[63] Gominet M Vadrot C Austruy G Darbord JC. Inactivation of Prion Infectivity by Ionizing Rays. Radiat Phys Chem (2007) 76:1760–2. doi: 10.1016/j.radphyschem.2007.02.099

[64] Tadesse A Eguale T Ashenafi H Tilahun G Ayana D. Enzymatic and Fecundity Evaluation of Fasciola Hepatica Exposed to Different Doses of γ- Irradiation in Ethiopian Sheep. Ethiop Vet J (2021) 25:85–114. doi: 10.4314/evj.v25i2.6

[65] Heidarieh M Hedayati Rad M Mirvaghefi AR Diallo A Mousavi S Sheikhzadeh N. Effect of Gamma Irradiation on Inactivation of Ichthyophthirius Multifiliis Trophonts and its Efficacy on Host Response in Experimentally Immunized Rainbow Trout (Oncorhynchus Mykiss). Turkish J Vet Anim Sci (2014) 38:388–93. doi: 10.3906/vet-1312-78

[66] Salehi B Motamedi-Sedeh F Madadgar O Khalili I Langroudi AGC Unger H. Analysis of Antigen Conservation and Inactivation of Gamma-Irradiated Avian Influenza Virus Subtype H9N2. Acta Microbiol Immunol Hung (2018) 65:163–71. doi: 10.1556/030.65.2018.025 29685054

[67] Ahmed S Ahmed B Mahmoud G Nemr W Abdel Rahim E. Comparative Study Between Formalin-Killed Vaccine and Developed Gamma Irradiation Vaccine Against Mannheimia Haemolytica in Rabbits. Turkish J Vet Anim Sci (2016) 40:219–24. doi: 10.3906/vet-1504-34

[68] Lulie S Alemayehu H Nuru A Abayneh T Eguale T. Immunogenicity and Protective Efficacy of Irradiated Salmonella Gallinarum Against Homologous Challenge Infection in Bovans Brown Chickens. Ethiop Vet J (2020) 24:123–38. doi: 10.4314/evj.v24i2.8

[69] Motamedi-Sedeh F Soleimanjahi H Jalilian AR Mahravani H Shafaee K Sotoodeh M. Development of Protective Immunity Against Inactivated Iranian Isolate of Foot-and-Mouth Disease Virus Type O/IRN/2007 Using Gamma Ray-Irradiated Vaccine on BALB/c Mice and Guinea Pigs. Intervirology (2015) 58:190–6. doi: 10.1159/000433538 26202581

[70] Sassu EL Kangethe RT Settypalli TBK Chibssa TR Cattoli G Wijewardana V. Development and Evaluation of a Real-Time PCR Panel for the Detection of 20 Immune Markers in Cattle and Sheep. Vet Immunol Immunopathol (2020) 227:1–10. doi: 10.1016/j.vetimm.2020.110092 32673891

[71] Kangethe RT Pichler R Chuma FNJ Cattoli G Wijewardana V. Bovine Monocyte Derived Dendritic Cell Based Assay for Measuring Vaccine Immunogenicity *in Vitro* . Vet Immunol Immunopathol (2018) 197:39–48. doi: 10.1016/j.vetimm.2018.01.009 29475505

[72] Fertey J Bayer L Grunwald T Pohl A Beckmann J Gotzmann G. Pathogens Inactivated by Low-Energy-Electron Irradiation Maintain Antigenic Properties and Induce Protective Immune Responses. Viruses (2016) 8:319. doi: 10.3390/v8110319 PMC5127033 27886076

[73] Fertey J Thoma M Beckmann J Bayer L Finkensieper J Reißhauer S. Automated Application of Low Energy Electron Irradiation Enables Inactivation of Pathogen- and Cell-Containing Liquids in Biomedical Research and Production Facilities. Sci Rep (2020) 10:12786. doi: 10.1038/s41598-020-69347-7 32732876 PMC7393095

[74] Fertey J Bayer L Kähl S Haji RM Burger-Kentischer A Thoma M. Low-Energy Electron Irradiation Efficiently Inactivates the Gram-Negative Pathogen Rodentibacter Pneumotropicus—A New Method for the Generation of Bacterial Vaccines With Increased Efficacy. Vaccines (2020) 8:1–10. doi: 10.3390/vaccines8010113 PMC7157226 32121656

[75] Moresco EMY LaVine D Beutler B. Toll-Like Receptors. Curr Biol (2011) 21:R488–93. doi: 10.1016/j.cub.2011.05.039 21741580

[76] Bode C Zhao G Steinhagen F Kinjo T Klinman DM. CpG DNA as a Vaccine Adjuvant. Expert Rev Vaccines (2011) 10:499–511. doi: 10.1586/erv.10.174 21506647 PMC3108434

