# Supplementary material for: Advances in Irradiated Livestock Vaccine Research and Production Addressing the Unmet Needs for Farmers and Veterinary Services in FAO/IAEA Member States
Source: Front Immunol. 2022 Mar 28;13:853874. doi: 10.3389/fimmu.2022.853874 (PMC8997582; doi:10.3389/fimmu.2022.853874)
Supplement: Supplementary file 1 [file Table_1.docx]

**Supplementary Table 1: Ongoing research projects funded through or done collaboratively by IAEA**

| **Project Type** | **Member State/s Involved** | **Main Counterpart Institute** | **Targeted Disease/ Pathogen/ Technology Developed** |
| --- | --- | --- | --- |
| **Technical Cooperation Projects (TCP)** | Tunisia | National Center for Nuclear Sciences and Technology (CNSTN), | Nodavirus in fish |
|  | Sri Lanka | University of Peradeniya | *Haemonchus contortus* in goats -field experiments |
| **Research Contracts Through Coordinated Research Projects (CRP)** | Bangladesh | Bangladesh Agricultural University | Avian mycoplasma |
|  | Indonesia | National Nuclear Energy Agency (BATAN) | *Streptococcus agalactiae* (mastitis) |
|  | Sri Lanka | University of Peradeniya | *Haemonchus contortus* in goats - R&D |
|  | Egypt | National Research Centre (NRC) | *Mannheimia haemolytica* and *Pasteurella multocida* |
|  |  |  |  |
|  | Iran | Nuclear Science and Technology Research Institute | Low Pathogenic Avian influenza (H9N2) |
|  | Sudan | Central Veterinary Research Laboratory Center | *Brucella meletensis* (Brucellosis) |
|  | Ethiopia | Addis Ababa University | *Salmonella gallinarum* (Fowl typhoid) |
|  | Ethiopia | Ethiopian Biotechnology Institute | *Pasteurella multocida* (Fowl cholera) |
|  | Kenya | Kenya Agricultural and Livestock Research Organization (KARLO) | Infectious bursal disease |
|  | Zambia | Central Veterinary Laboratory | *Theileria parva* |
| **Research Agreements Through Coordinated Research Projects (CRP)** | Italy | Italian health authority and research organization for animal health and food safety (IZSVe) | Low Pathogenic Avian influenza (H9N2) |
|  | Germany | Fraunhofer Institute for Cell Therapy and Immunology IZI | E-beam technology for vaccine development |
| **Collaborative Research Programs with Animal Production and Health Laboratory** | Austria | The Austrian Agency for Health and Food Safety (AGES) | Porcine Reproductive and Respiratory Syndrome (PRRS) |
|  | Austria | University of Veterinary Medicine Vienna | *E. coli* in chicken |
|  | Austria | University of Natural Resources and Life Sciences (BOKU) | Irradiation of probiotic bacteria as vaccine adjuvants |
|  | Germany | Friedrich Loeffler Institute (FLI) | African Swine Fever |
